# Inulin-Type Fructan Supplementation of 3- to 6-Year-Old Children Is Associated with Higher Fecal *Bifidobacterium* Concentrations and Fewer Febrile Episodes Requiring Medical Attention

**DOI:** 10.1093/jn/nxy120

**Published:** 2018-07-03

**Authors:** Szimonetta Lohner, Viktória Jakobik, Krisztina Mihályi, Sara Soldi, Sotirios Vasileiadis, Stephan Theis, Manuela Sailer, Carolin Sieland, Károly Berényi, Günther Boehm, Tamás Decsi

**Affiliations:** 1 Department of Paediatrics, Clinical Center of the University of Pécs; 2 Department of Public Health Medicine, Medical School, University of Pécs, Pécs, Hungary; 3 Advanced Analytical Technologies Srl, Fiorenzualo d'Arda (Pc), Italy; 4 Department of Biochemistry & Biotechnology, University of Thessaly, Larissa, Greece; 5 Beneo-Institute, Obrigheim, Germany; 6 Nutritional Science Consulting, Leipzig, Germany

**Keywords:** prebiotics, randomized controlled trial, double-blind method, placebo, children, prevention, respiratory tract infections, gastrointestinal infections, stool consistency, microbiota composition

## Abstract

**Background:**

Inulin-type fructans used in formula have been shown to promote microbiota composition and stool consistency closer to those of breastfed infants and to have beneficial effects on fever occurrence, diarrhea, and incidence of infections requiring antibiotic treatment in infants.

**Objectives:**

The primary study aim was to explore whether prophylactic supplementation with prebiotic fructans is able to influence the frequency of infectious diseases in kindergarten children during a winter period. A secondary objective was to ascertain the effect on the intestinal microbiota.

**Methods:**

142 boys and 128 girls aged 3–6 y were randomly allocated to consume 6 g/d fructans or maltodextrin for 24 wk. At baseline, stool samples were collected for microbiota analysis and anthropometric measurements were made. During the intervention period diagnoses were recorded by physicians, whereas disease symptoms, kindergarten absenteeism, dietary habits, and stool consistency were recorded by parents. Baseline measurements were repeated at wk 24.

**Results:**

In total 219 children finished the study. Both the relative abundance of *Bifidobacterium* (*P* < 0.001) and that of *Lactobacillus* (*P* = 0.014) were 19.9% and 7.8% higher, respectively, post data normalization, in stool samples of children receiving fructans as compared with those of controls at wk 24. This was accompanied by significantly softer stools within the normal range in the prebiotic group from wk 12 onwards. The incidence of febrile episodes requiring medical attention [0.65 ± 1.09 compared with 0.9 ± 1.11 infections/(24 wk × child), *P* = 0.04] and that of sinusitis (0.01 ± 0.1 compared with 0.06 ± 0.25, *P* = 0.03) were significantly lower in the prebiotic group. The number of infectious episodes and their duration reported by parents did not differ significantly between the 2 intervention groups.

**Conclusions:**

Prebiotic supplementation modified the composition of the intestinal microbiota and resulted in softer stools in kindergarten-aged children. The reduction in febrile episodes requiring medical attention supports the concept of further studies on prebiotics in young children. This trial was registered at clinicaltrials.gov as NCT03241355.

## Introduction

Acute infections are a common cause of health care–seeking behavior among parents of preschool children (1); they significantly increase medical expenses and workday losses (2) and therefore can be considered a public health problem worldwide. Effective preventive interventions can have huge benefits for health care systems in terms of reducing burden and costs.

There is consensus that the human gut microbiota actively interacts with the host, influencing the function of the intestine, metabolism, and immune system (3–5). Acknowledged prebiotics (3, 6) like galacto-oligosaccharides and inulin-type fructans, such as inulin and oligofructose, are used in infant formulae in order to mimic the prebiotic effect of human milk oligosaccharides with respect to modulating gut microbiota composition including higher *Bifidobacterium* abundance. Positive effects have been demonstrated, especially for intestinal function and physiology (e.g., improved stool consistency and frequency) and the immune system (e.g., reduced risk of infections and incidence of allergic symptoms) (7–9). Moreover, the production of SCFAs during the fermentation process can affect the immune function via several mechanisms including the acidification of the colonic lumen and stimulation of immune cells within the gut-associated lymphoid tissues (5). The composition of prebiotic fructans including chain-length distribution could influence the fermentation characteristics along the intestine, and various fermentation patterns could synergistically influence systemic physiological processes such as the immune system (10, 11).

Because the development of the immune system, which is influenced by the gut microbiota composition, continues also beyond the first year of life, prebiotics might have important health effects also during the following years of life. However, studies investigating immune-modulating and infection-preventive effects of prebiotics in children aged ≥2 y are limited (12).

We presumed that, as during infancy, the gut microbiota can be influenced by supplementation of a prebiotic Orafti inulin-type fructan product with shorter and longer chains in kindergarten-aged children. We further postulated that this prebiotic effect stimulates the immune system resulting in reduced incidence of infections.

The main aim of the present study was to explore whether prophylactic dietary supplementation with 6 g/d of prebiotic inulin-type fructans in healthy children aged 3–6 y over a 6-mo period could beneficially influence the gut microbiota composition, resulting in a higher relative abundance of *Bifidobacterium* and *Lactobacillus*, and decrease the frequency of infectious disease episodes.

## Methods

### 

#### Study details

The present study was a randomized, parallel, double-blind, placebo-controlled trial (NCT03241355). The flow diagram of the study is presented in **Supplemental Figure 1**. Children aged 3–6 y on enrolment were recruited continuously in 22 kindergartens in the region of Pécs, Hungary between September 1 and October 15, 2013. The explorative nature of the study did not allow a sample size determination. In order to obtain statistically representative results, we estimated a number of 100 completers per group. Assuming a dropout rate of about 25%, we aimed to recruit ≥125 children per group.

#### Subjects

Eligible children had to meet the following criteria: *1)* >3 y but <7 y of age on pre-examination, *2)* healthy on inclusion, and *3)* attending a kindergarten. Exclusion criteria were: congenital disease or malformation influencing the gastrointestinal system, immunodeficiency, food intolerance, food allergy, or metabolic disorder requiring a special diet, regular (>3 times per wk) consumption of products or food supplements containing prebiotics or probiotics, antibiotic or laxative treatment, and/or any infectious disease within 14 d at the time of pre-examination. Children were included in the study after their parents had provided written informed consent.

#### Recruitment strategy

Children were continuously recruited from September 1, 2013 until October 15, 2013. Recruitment sites were kindergartens within the region of Pécs, Hungary. Advertisements for subject recruitment were posted in kindergartens, and presentations about the study aims were held at parent meetings. Interested parents or guardians of children were subsequently provided with information material about the purpose of the study and were asked to return for the pre-examination visit, where children were checked for the inclusion and exclusion criteria.

#### Randomization

Children found suitable for inclusion in the study were randomly assigned with a 1:1 allocation ratio to the prebiotic or the placebo group. The list of random assignments was generated using the website www.randomization.com by an investigator not participating in the implementation of the trial. A block randomization was carried out with a block size of 4, where the 4 strata were as follows: boys receiving prebiotics, boys receiving placebo, girls receiving prebiotics, and girls receiving placebo. Sequentially numbered study products were chronologically allocated to the corresponding subject according to their time of study entry. The study personnel (responsible for participant enrolment and group assignment) and the parents were unaware of the group allocation. The package of all products was identical; identification was not possible based on the randomization numbers.

#### Intervention

The intervention period lasted for 24 wk during winter in order to cover the whole season with high infection rates. Children received the supplement once daily at the same time, mixed in their food or drink, while their basal nutrition remained unchanged. The prebiotic group received 6 g of an Orafti inulin-type fructan product with shorter and longer chains [degree of polymerization (DP) ≥11 approx. 25–30% (on g/100 g DM), average DP of about 7–8] daily, whereas the placebo group received maltodextrin in the same dosage. The daily dosage of 6 g was chosen to ensure a prebiotic modulation of the gut microbiota and to have an intake amount that is well tolerated.

#### Outcomes

Frequency of infectious disease episodes was defined as the primary outcome. Secondary outcomes included duration of infectious disease episodes, frequency of overt diarrheal episodes and development of liquid stools during antibiotic treatment, days of absence from daycare due to infectious disease and/or symptoms of diarrhea after antibiotic treatment, frequency of antibiotic treatments, number of physician’s consultations for symptoms of an infectious disease, and hospitalization days due to infectious disease. Stool consistency, microbiota composition (including total bacteria, relative abundance of *Bifidobacterium* and *Lactobacillus*, and relative abundance of pathogenic microorganisms [*Clostridium difficile*, *Clostridium perfringens*, Enterobacteriaceae]), and fecal pH were assessed as well. The incidence of gastrointestinal discomfort as well as anthropometric parameters and tolerance and acceptance of the study product were recorded as safety parameters. Parameters describing the family environment (e.g., smoking or pets in the household, number of siblings) were recorded as potential confounding variables.

#### Assessment of outcomes

The *incidence of acute respiratory, gastrointestinal*, *and urinary infections* was recorded via case report forms (CRFs) and diaries filled in by the parents and questionnaires filled in by the physicians. Parents continuously had to fill in a study diary, where they had to register all infectious disease symptoms during the study period. Respiratory symptoms included, e.g., runny nose, repetitive sneezing, cough, sore throat; as well as gastrointestinal symptoms including vomiting and/or diarrhea. Fever was defined as peak rectal temperature of ≥38.5°C or axillar temperature of ≥38.0°C. Diarrhea was defined as ≥3 loose-to-watery stools (Type 7 on the Bristol Stool Scale) in a 24-h period that exceeded the child's usual daily stool frequency and was perceived by the parents as diarrhea.

Every 6 wk, study diaries were collected and new diaries were handed out. Parents had to register the duration of infections, the number of physician visits, the days the child was hospitalized, the days the child was absent from the kindergarten, any prescribed antibiotics or other medication, and all other observations they found important. The diary was also used to ascertain the number and duration of febrile episodes.

An infectious disease episode was defined as a period with symptoms of illnesses indicating a viral or bacterial infection (e.g., cough, wheeze, sneeze, runny nose, sore throat, vomiting, diarrhea) as recorded in the diaries of the parents. The episode was considered finished if the child ceased to exhibit the respective symptoms. Another episode was considered as a new episode if the symptoms occurred ≥3 d after recovery from a previous episode.

All local pediatricians (hereinafter called physicians) working in Pécs were informed about the study and all were willing to cooperate. Physicians were asked to record the diagnosis and the prescribed treatment at every visit. In cases when the parents reported a visit to the physician in the study diary, but the physician's CRF was missing for any reason, physicians were contacted directly to get information about the diagnosis and prescribed treatment retrospectively.

Acute upper respiratory infections included common cold (rhinitis acuta, rhinosinusitis acuta), sinusitis maxillaris (acute bacterial sinusitis), pharyngitis acuta/tonsillitis acuta/tonsillopharyngitis acuta, croup (laryngotracheobronchitis/laryngotracheitis), and acute infectious laryngitis. Acute lower respiratory tract infections included acute bronchitis/acute tracheobronchitis and pneumonia. The diagnoses were set by all pediatricians based on the current Hungarian medical guidelines.

*Dietary habits* of the participating children were evaluated via a FFQ filled in by the parents 3 times during the study, covering a period of 3 d including 2 working days and 1 weekend day. Energy and nutrient intakes were calculated with the use of specially developed software: NutriComp Étrend Sport (13, 14).

*Parameters describing the family environment* (number of children in household, furry pets in household, smoking and education level of parents) were collected via a questionnaire filled in by the parents at the start of the study.

*Stool consistency* was evaluated by the parents based on the Bristol Stool Chart (a medical tool designed to classify the form of human feces into 7 categories, from severe constipation to severe diarrhea) 4 times during the whole study period (on study wk 6, 12, 18, and 24), each time for 7 d. For each week the mean stool consistency was calculated.

*Safety parameters* included anthropometric parameters, incidence of gastrointestinal discomfort, as well as tolerance and acceptance of the study products. Parents had to answer the following questions in the CRFs: “Was the supplement generally well tolerated by the child?”, “Did you notice any negative effects which you think are related to the consumption of the supplement? If yes, please specify”, and “Has your child complained about abdominal discomfort?”

The *microbiological investigation* included a quantification of total bacteria, the analysis of the relative abundance of *Bifidobacterium* and *Lactobacillus* as markers for a beneficial composition, as well as of pathogens, such as *C. difficile*, *C. perfringens*, and Enterobacteriaceae. Approximately 2 g of feces was collected at study start and end, refrigerated at home at 4°C and collected and transported by study personnel in travel freezers to the study center for storage at –80°C within 24 h. Fecal samples were shipped on dry ice to Advanced Analytical Technologies, Fiorenzuola d'Arda (PC), Italy for analysis. Fecal samples were thawed and divided into 2 aliquots for DNA extraction and pH measurement. Fecal bacterial DNA was extracted through the use of a FastDNA SPIN Kit and FastPrep Instrument (MP Biomedicals, Santa Ana, CA). The extracted DNA was quantified via the picogreen method of the Quant-iT™ HS ds-DNA assay kit in a Qubit™ fluorometer (Invitrogen, Carlsbad, CA). DNA extracts were diluted up to 5 ng/µL for reducing template amount–associated PCR biases. The proper volume of RT-PCR reagents was freshly prepared for each RT-PCR analytical session, according to the number of samples in duplicates, for a final reaction volume of 25 μL, including also a positive control. The amplification mixtures were prepared through the use of the RealMasterMix Sybr Rox 2.5X (5 PRIME, FisherScientific), according to the manufacturer's instructions. Different protocols and primer pairs were applied for the detection of the bacteria *Bifidobacterium, Lactobacillus, C. perfringens, C. difficile*, and for the family Enterobacteriaceae as previously documented (15–18). Thermal cycling protocols were performed as provided in the cited publications. For pH measurement an aliquot of each fecal sample was diluted 1:10 with a saline solution and mixed carefully up to complete dissolution. The pH was then measured after appropriate calibration and following the recommendations of the instrument's supplier (Laboratory pHmeter GLP 21, Crison).

#### Monitoring of compliance

Parents were contacted every 6 wk; at these occasions study diaries and CRFs were collected and reviewed. If the parents had difficulties with filling in the questionnaires, help was provided by the study personnel. Moreover, parents could reach study personnel and request help on each and every day during the whole intervention period. CRFs and diaries were regularly evaluated and data were recorded parallel with the intervention. If CRFs and diaries were not clearly filled in or data were missing, parents were contacted. Children whose parents permanently couldn't be contacted or did not cooperate in the required extent to provide data on the infection episodes of their child were excluded from the study. In the CRF questions were asked about the time and method of supplement consumption and the number of days when the supplement was not consumed.

Data of children whose parents withdrew from participation with or without explanation, and of those with <30% missing data or who discontinued their specific feeding regimen before study termination, were excluded from the study and subsequent statistical data evaluation.

#### Statistical analysis

Statistical analyses of the data were performed based on the per-protocol population with the use of the Statistical Package for the Social Sciences (IBM SPSS version 22.0). Continuous variables are reported as means and SDs, or medians and IQRs, and categorical variables are reported as numbers or percentages. For quantitative variables a Mann-Whitney test was used after rejecting the null hypothesis of the Shapiro-Wilk test of normal distribution; for variables expressed as percentages, a Pearson chi-square test was used. Anthropometric measurements and incidence and duration of diseases were also evaluated with the Mann-Whitney test. To compare the proportions of children with specific acute disease incidences, as well as the antibiotic prescription rates of both groups, a Pearson chi-square test was used. A multivariate logistic regression analysis was conducted to reveal the factors that influenced the number of recurrent infections.

Microbial data are normalized via Box-Cox transformed values. Data are expressed as ratio of taxon to total bacteria and displayed as mean ± SD. Statistical comparison was performed via Student's *t* test.

Results with a significance level of *P* < 0.05 were considered statistically significant.

#### Ethical approval

The study was approved by the Regional Research Ethics Committee of the Medical Center of Pécs (Docet No.: 4829/2013) and the Scientific and Research Ethics Committee of the Medical Research Council, Budapest, Hungary (40564–3/2013/EKU (477/2013)) and permitted by the Policy Administration Services of Public Health, Government Office of Baranya County (Docet No.: BAR/006/94–7/2013).

## Results

### 

**FIGURE 1:**
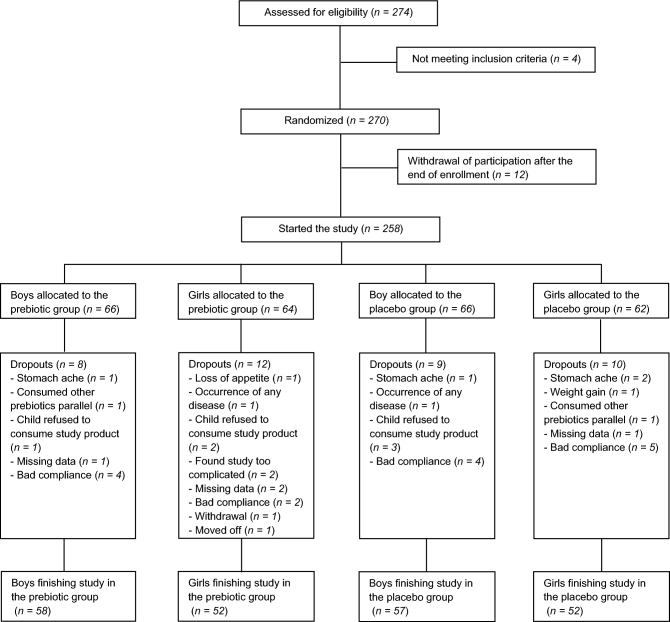
Flowchart of participants through the randomized controlled double-blind explorative study investigating the effect of prebiotic inulin-type fructans on acute infectious diseases in children.

In September and October 2013, 274 children were assessed for eligibility, of which 258 started the study (**Figure 1**): 130 in the prebiotic group and 128 in the placebo group. There was no statistically significant difference either in dropout numbers or in gender distribution of the children completing the study in the prebiotic or placebo group (Figure 1). Only those children who completed the study (*n* = 219) were analyzed.

#### Characterization of the study groups

Baseline characteristics of children are presented in **Table 1**. There were no significant differences in these characteristics or in daily energy and macronutrient intake in the 2 groups over the course of the study (data not presented). Daily fiber intake over the course of the study did not differ either (data not presented).

**TABLE 1:** Baseline characteristics of the children enrolled in the study^1^

	Prebiotic	Placebo
	group (*n* = 110)	group (*n* = 109)
Gender, *n* boys/*n* girls	58/52	57/52
Age at enrollment, y	4.93 (1.54)	4.97 (1.72)
Weight at enrollment, kg	18.0 (4.50)	18.0 (6.00)
Height at enrollment, m	1.1 (0.13)	1.1 (0.12)
Children in the household	2.0 (1.00)	2.0 (1.00)
Single-parent family	21.5	22.6
Family with furry pets in household	38.9	34.0
Smoking mother	26.9	18.9
Smoking father	38.5	30.6

^1^Values are percentages or medians (IQRs).

#### Fecal microbiota composition and pH measurement

**FIGURE 2:**
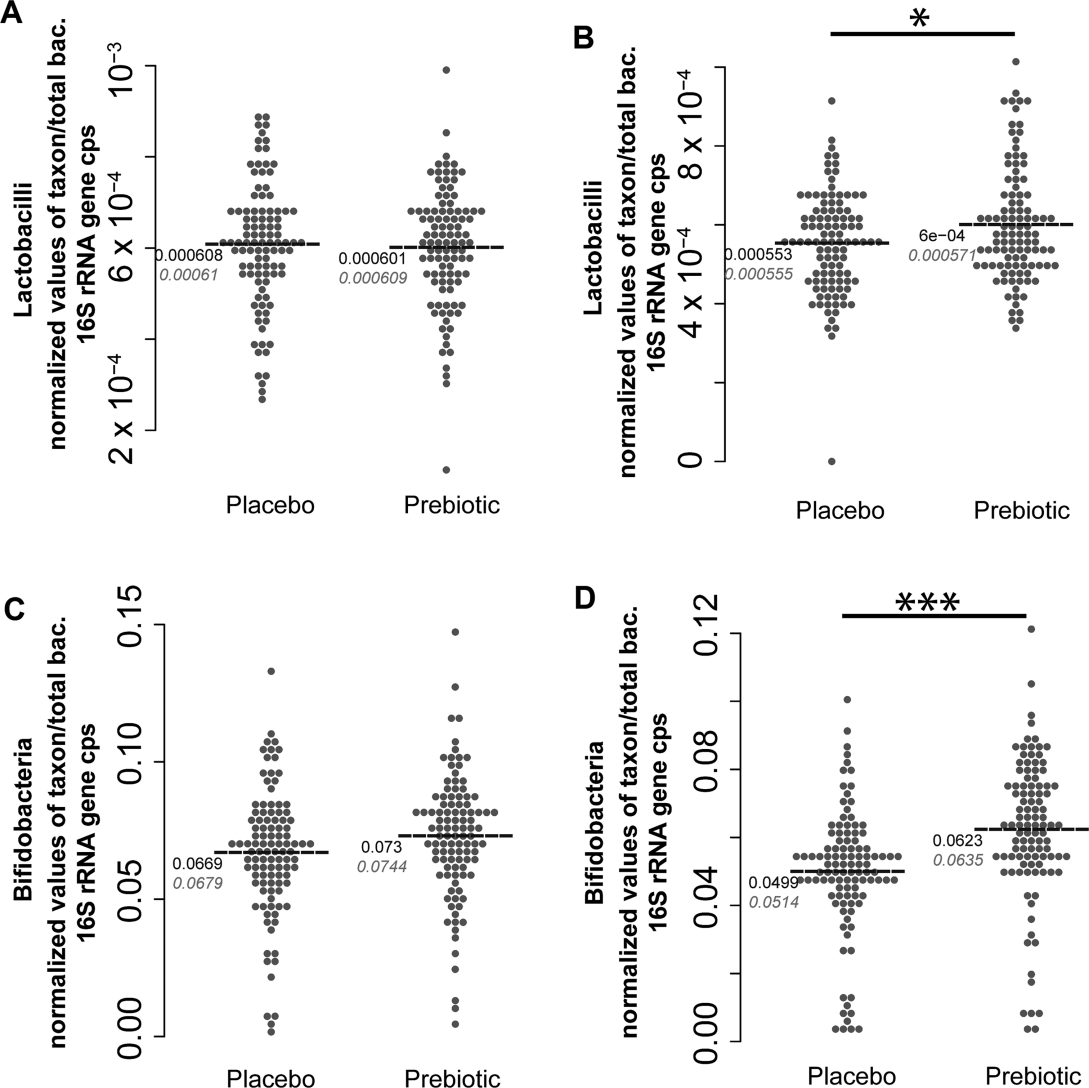
*Lactobacillus* (A, B) and *Bifidobacterium* (C, D) at baseline (A, C) and wk 24 (B, D) in healthy children aged 3–6 y supplemented with inulin-type fructans or placebo. Values are normalized via Box-Cox transformations and expressed as relative to total bacteria 16S rRNA gene copies, *n* = 109 (placebo) or 110 (fructans). Black values represent means. Gray values represent medians. Group comparison was performed via Student's *t* test. * *P* < 0.05, *** *P* < 0.001.

Fecal microbiota composition at baseline did not differ between the 2 groups (**Figure 2**, **Table 2**). Intake of the prebiotic product resulted in specific changes to the microbiota composition. Relative abundance of *Bifidobacterium* was significantly (*P* = 1.228 × 10^−05^) higher in children receiving the prebiotic product than in controls at study end. Similarly, relative abundance of *Lactobacillus* at study end was significantly higher in the prebiotic as compared with the placebo group (*P* = 0.014) (Figure 2). There was no difference between the groups for total bacteria or the relative abundance of *Clostridia* species and Enterobacteriaceae populations (Table 2). Fecal pH tended to be lower in the prebiotic group compared with the placebo group throughout the study (*P* = 0.11).

**TABLE 2:** Abundance of total bacteria and relative abundance of *Clostridium perfringens*, *Clostridium difficile*, and Enterobacteriaceae at baseline and at wk 24, in both the prebiotic and the placebo groups^1^

	Prebiotic group (*n* = 110)		Placebo group (*n* = 109)	
	Baseline	Wk 24	Baseline	Wk 24
	(mean ± SD)	(mean ± SD)	(mean ± SD)	(mean ± SD)
Total bacteria	9.4 × 10^10^ ± 2.2 × 10^10^	9.8 × 10^10^ ± 1.7 × 10^10^	9.5 × 10^10^ ± 1.9 × 10^10^	9.7 × 10^10^ ± 1.8 × 10^10^
*C. perfringens*: total	7.2 × 10^−5^ ± 6.1 × 10^−6^	5.4 × 10^−5^ ± 7.1 × 10^−6^	7.2 × 10^−5^ ± 6.1 × 10^−6^	5.4 × 10^−5^ ± 7.8 × 10^−6^
*C. difficile*: total	2.3 × 10^−6^ ± 1.2 × 10^−8^	1.3 × 10^−6^ ± 1.4 × 10^−8^	2.3 × 10^−6^ ± 1.3 × 10^−8^	1.2 × 10^−6^ ± 1.4 × 10^−8^
Enterobacteriaceae:total	0.0017 ± 0.00080	0.00157 ± 0.00068	0.00178 ± 0.00099	0.00152 ± 0.00080

^1^Values are normalized via Box-Cox transformation and in the cases of *C. perfringens*, *C. difficile*, and Enterobacteriaceae are expressed as ratio to total bacteria 16S rRNA gene copies. Group comparison was performed via Student's *t* test.

#### Health-related outcomes

**Table 3** presents the incidence of infections diagnosed by a physician during the 24-wk intervention period. The number of febrile episodes requiring medical attention was lower in the prebiotic group [infections/(24 wk × child), mean ± SD: 0.65 ± 1.09] compared with the placebo group [0.90 ± 1.11 infections/(24 wk × child), *P* = 0.04], as was the incidence of sinusitis [0.01 ± 0.1 compared with 0.06 ± 0.25 infections/(24 wk × child), *P* = 0.03].

**TABLE 3:** Number of infections diagnosed by a physician in healthy children aged 3–6 y during 24 wk of an intervention with inulin-type fructans (*n* = 110) as compared with placebo (*n* = 109)^1^

	Prebiotic infections,	Prebiotic infections,	Placebo infections,	Placebo infections,	
Diagnosis	*n*/24 wk	*n*/(24 wk × child)^2^	*n*/24 wk	*n*/(24 wk × child)^2^	*P*
Overall infections requiring physician's consultation	157	1.43 ± 1.32	184	1.69 ± 1.50	0.24
Febrile infections requiring physician's consultation	71*	0.65 ± 1.09	98	0.90 ± 1.11	0.04
Tonsillopharyngitis acuta	110	1.00 ± 0.99	123	1.13 ± 1.06	0.39
Sinusitis maxillaris	1*	0.01 ± 0.1	7	0.06 ± 0.25	0.03
Laryngitis acuta	0	0.00 ± 0.00	1	0.01 ± 0.1	0.32
Otitis media	10	0.09 ± 0.37	14	0.13 ± 0.39	0.24
Bronchitis acuta	15	0.14 ± 0.44	17	0.16 ± 0.43	0.54
Pneumonia	2	0.02 ± 0.13	3	0.03 ± 0.16	0.64
Gastroenteritis acuta	18	0.16 ± 0.42	19	0.17 ± 0.43	0.83
Urinary tract infection	1	0.01 ± 0.10	0	0.00 ± 0.00	0.32
Total number of URTIs	111	1.01 ± 1.02	131	1.20 ± 1.14	0.26
Total number of LRTIs	17	0.15 ± 0.43	20	0.18 ± 0.47	0.55

^1^Values are total numbers and means ± SDs. *Different from placebo, *P* < 0.05. LRTIs, lower respiratory tract infections; URTIs, upper respiratory tract infections.

^2^Incidence of infectious diseases was evaluated with the Mann-Whitney test.

There was no significant difference in the cumulative incidence of recurrent infections (incidence of having ≥2 infection episodes during the 24-wk study period) of any kind (39.1% compared with 45.9%) and in the cumulative incidence of recurrent upper respiratory tract infections (23.6% compared with 30.3%) between the prebiotic and placebo groups.

There was no significant difference between the groups in the number of infectious episode symptoms reported by the parents during the 24-wk intervention period (**Table 4**).

**TABLE 4:** Number of infectious episodes, reported by the parents of healthy children aged 3–6 y consuming either inulin-type fructans (*n* = 110) or placebo (*n* = 109) for 24 wk^1^

	Prebiotic episodes,	Prebiotic episodes,	Placebo infections,	Placebo episodes,	
	*n*/24 wk	*n*/(24 wk × child)	*n*/24 wk	*n*/(24 wk × child)	*P*
Episodes with respiratory symptoms^2^	311	2.83 ± 1.89	307	2.82 ± 1.87	0.84
Episodes with gastrointestinal symptoms^3^	36	0.33 ± 0.65	39	0.36 ± 0.66	0.47
Fever episodes	78	0.71 ± 1.01	81	0.81 ± 0.74	0.83
Infections requiring antibiotic prescription	71	0.65 ± 1.04	76	0.70 ± 0.91	0.37
Infections requiring hospitalization	0	0.00 ± 0.00	2	0.02 ± 0.13	0.15

^1^Values are total numbers and means ± SDs.

^2^Respiratory symptoms included runny nose, repetitive sneezing, cough, and sore throat.

^3^Gastrointestinal symptoms included days with diarrhea and/or vomiting.

Based on the parents’ questionnaires there was no significant difference in the total number of infection days, febrile days, total days of antibiotic treatment, total days of absence from day-care, and the total number of days spent in hospital due to an infectious disease between the 2 groups (**Table 5**).

**TABLE 5:** Total number of infection days, days of absence from daycare due to infection, and hospitalization days in healthy kindergarten-aged children consuming a dietary supplement with either inulin-type fructans or placebo for 24 wk, reported by the parents

	Prebiotic days,	Placebo days,	
	*n*/24 wk (*n* = 110)	*n*/24 wk (*n* = 109)	*P*
Days of overall infections	2550	2324	0.66
Febrile days	199	202	0.73
Days of antibiotic treatment	435	485	0.32
Absence from daycare	1040	1054	0.52
Hospitalization	2	10	0.17

There was no significant difference between the 2 groups in the duration of either infections with respiratory tract symptoms (e.g., runny nose, repetitive sneezing, cough, sore throat; 7.92 ± 3.39 compared with 7.52 ± 3.50 d, mean ± SD, prebiotic compared with control group) or gastrointestinal disease symptoms (days with diarrhea and/or vomiting; 4.83 ± 1.10 compared with 5.50 ± 2.03, mean ± SD).

#### Analysis of different influential factors on disease incidence

To reveal the factors that influenced the number of recurrent infections we performed a multivariate logistic regression analysis. This analysis identified 2 factors having significant influence on recurrent infection episodes: both younger children and children from families with only 1 child had significantly higher numbers of recurrent infection episodes of any kind (**Table 6**).

**TABLE 6:** Multivariate regression analysis of recurrent infection episodes in healthy kindergarten-aged children consuming a dietary supplement with either inulin-type fructans (*n* = 110) or placebo (*n* = 109) for 24 wk

	OR (95% CI)
Furry pets in the household	0.58 (0.31, 1.09)
Number of children in the family	0.69 (0.51, 0.94)
Study group (prebiotic vs. control)	0.73 (0.41, 1.30)
Age	0.73 (0.56, 0.96)
Gender	0.83 (0.46, 1.48)
Mean daily vitamin D intake, μg/d	0.85 (0.49, 1.48)
Mean daily fiber intake, g/d	0.97 (0.87, 1.07)
Probiotic supplement intake, times/study period	0.98 (0.96, 1.00)
Mean daily vitamin C intake, mg/d	1.00 (0.97, 1.01)

#### Safety parameters

The main safety parameter analyzed was gastrointestinal discomfort. There was no significant difference between the groups in the number of children with gastrointestinal discomfort either at the beginning or at the end of the study. In the first 6-wk period 16 children consuming prebiotics and 15 children consuming placebo had abdominal discomfort; during the fourth 6-wk period the number of children with abdominal complaints was 8 in the prebiotic and 11 in the placebo group. There was 1 dropout in the prebiotic and 3 in the placebo group due to stomachache (Figure 1). There were no other signs of intolerance in either of the 2 groups.

#### Stool parameters

There was no significant difference in stool consistency after 6 wk supplementation (score on the Bristol Stool Scale was 3.17 [1.18] in the prebiotic and 3.00 [1.18] in the placebo group; median [IQR]), whereas after 12 wk (3.64 [1.04] compared with 3.16 [1.08]; *P* = 0.003), 18 wk (3.28 [1.14] compared with 3.00 [0.88]; *P* = 0.004), and 24 wk (3.33 [1.35] compared with 3.00 [1.00]; *P* = 0.008) of the intervention, stools were significantly softer in the prebiotic group compared with those in the placebo group.

## Discussion

This randomized controlled double-blind explorative study, conducted in 219 healthy children aged 3–6 y during the winter period, found significantly lower numbers of febrile episodes requiring physician's consultation and fewer episodes of sinusitis in children consuming 6 g/d of prebiotic Orafti inulin-type fructan product with shorter and longer chains for 24 wk as compared with children consuming maltodextrin as placebo. In the prebiotic group an increase in the relative abundance of *Bifidobacterium* and constant levels of *Lactobacillus* could be observed whereas in the placebo group the relative abundance of *Bifidobacterium* and *Lactobacillus* decreased. These changes resulted in significantly higher levels of *Bifidobacterium* and *Lactobacillus* in the prebiotic group at the end of the intervention. This was accompanied by significantly softer stool consistency during the second half of the study. The findings indicate that the tested Orafti inulin-type fructan product with shorter and longer chains has an effect on microbiota composition and also might influence health outcomes related to immunity in children aged 3–6 y comparable with effects reported in infants (7–9, 12, 19, 20).

In the present study, both the prebiotic supplement and the placebo were well tolerated by children in a daily dose of 6 g, as demonstrated by the low dropout rate due to abdominal complaints and the absence of adverse events. This is in line with findings of previous studies reporting that chicory-derived inulin-type fructans are safe and well tolerated by infants and children aged 2–5 y (7, 19, 21, 22).

Inulin-type fructans used in infant and follow-on formula have been shown to have a bifidogenic effect and to soften stools, promoting a microbiota composition and stool characteristics that are closer to those of breastfed infants (7, 9, 20). The present study provides evidence that children aged 3–6 y—an age group for which data on prebiotic effects were lacking thus far—may also benefit from regular prebiotic inulin-type fructans intake. The positive impact on stool consistency observed (i.e., softer stool consistency measured on the Bristol Stool Scale) has been reported recently also in constipated but otherwise healthy children aged 2–5 y (21). The reduction of *Bifidobacterium* and *Lactobacillus* in the control group and the restoring/increase of *Bifidobacterium* and *Lactobacillus* in the prebiotic-supplemented group may have further implications for health later in life as also put forward by Cheng et al. (23). For instance, elevated *Bifidobacterium* levels in early life are regarded as being protective against weight gain and neuropsychiatric disorders in later life (24–26).

The modulation of the gut microbiome is an evolving strategy to improve human health. Prebiotics are recognized for their ability to manipulate the host microbiota of the gastrointestinal tract and their activity to the benefit of the host (27). Their fermentation by the gut microbiota leads to production of SCFAs, lower amounts of ammonia, and overall decreased pH values (28). This may have immunomodulatory effects and enhance barrier function (5). Comparable processes could be assumed to be operative in the present study. Furthermore, fructans may support immunity, partially in a microbiota-independent and in a chain-length–dependent manner, by directly interacting with immune cells (29–31).

Infant studies have already showed that prebiotics of nonmilk origin can mimic the prebiotic effect of breastfeeding and, consequently, have positive effects on the postnatal development of the immune system (4, 10). Previous studies with inulin-type fructans compared to placebo found a decreased number of fever episodes accompanying cold symptoms in infants aged 4–24 mo (32) and fewer episodes with fever or diarrhea and a lower number of infectious diseases requiring antibiotic treatment in children aged 7–19 mo (33), suggesting that inulin-type fructans are able to positively modulate the performance of the immune system. The present study found a significant reduction of febrile episodes requiring a physician's consultation in the prebiotic group. However, according to the parents’ reports there was no significant difference between the 2 intervention groups regarding the number of acute infectious episodes and their duration. This might be explained by the circumstances listed hereinafter as limitations of this study.

One limitation may be the lack of predefined differential diagnosis criteria that physicians could follow when investigating children. Similarly, it was not specified for parents in which cases they have to visit their physician. However, randomization should have limited the between-group differences.

The strengths of the present study include the randomized double-blind design, the close follow-up of children during the study, and the regular contact with their parents. Dropout rates were similar between the 2 groups (15.38% in the prebiotic and 14.84% in the placebo group) and rather low for this type of study. All these factors minimize the risk of bias. No other prebiotic/probiotic products were allowed to be consumed during the study; moreover, occasional consumption of other prebiotics or probiotics was strictly recorded via parents’ CRFs and hence interfering effects of other prebiotics/probiotics could be excluded.

A further strength of our study is that the numbers of infectious episodes were evaluated from 2 different points of view: firstly, parents recorded the number of days with different symptoms they had observed and associated with infectious diseases; and secondly, common infectious diseases diagnosed by physicians were also recorded. Parents’ health care–seeking behavior was not heavily influenced by the study; hence, the results of this study can be easily adapted to a real-life situation and might be relevant for prebiotic strategies in praxis.

Large conclusive studies are thus justified to examine in further detail whether the favorable modulation of the gut microbiota in preschool children also results in beneficial effects on their immune system, and whether the moderate, but significant effects seen in this study can be interpreted as signs of this beneficial immune-modulatory effect.

In conclusion, prebiotic supplementation with Orafti inulin-type fructan product with shorter and longer chains in a dose of 6 g/d for 24 wk significantly modified the levels of *Bifidobacterium* and *Lactobacillus* and resulted in softer stool consistency in children aged 3–6 y as compared with maltodextrin. The significant reduction in febrile episodes requiring a physician's consultation in the prebiotic group supports the concept of further studies on prebiotic supplementation in young children.

## Supplementary Material

Supplemental File

## Notes

Supported by BENEO GmbH, Mannheim, Germany, a member of the Suedzucker Group.

Author disclosures: ST, MS, and CS are/were employees of BENEO / Suedzucker Group. GB has acted as a consultant for BENEO / Suedzucker Group. TD received research funding from the National Research, Development and Innovation Office, Hungary (grant OTKA 120193) to conduct a study on the role of prebiotics in preventing childhood infections. SL, VJ, KM, SS, SV, and KB: no conflicts of interest.

The study results and data contained in the publication have been developed for BENEO GmbH. BENEO GmbH reserves the exclusive right to use the results and data for possible Health Claim requests.

Supplemental Figure 1 is available from the “Supplementary data” link in the online posting of the article and from the same link in the online table of contents at https://academic.oup.com/jn/.

Abbreviation used:

CRFs: case report forms
