# Supplementary material for: Inulin-Type Fructan Supplementation of 3- to 6-Year-Old Children Is Associated with Higher Fecal Bifidobacterium Concentrations and Fewer Febrile Episodes Requiring Medical Attention
Source: J Nutr. 2018 Jul 3;148(8):1300–8. doi: 10.1093/jn/nxy120 (PMC6074834; doi:10.1093/jn/nxy120)
Supplement: Supplemental File [file nxy120_supplemental_file.pdf]

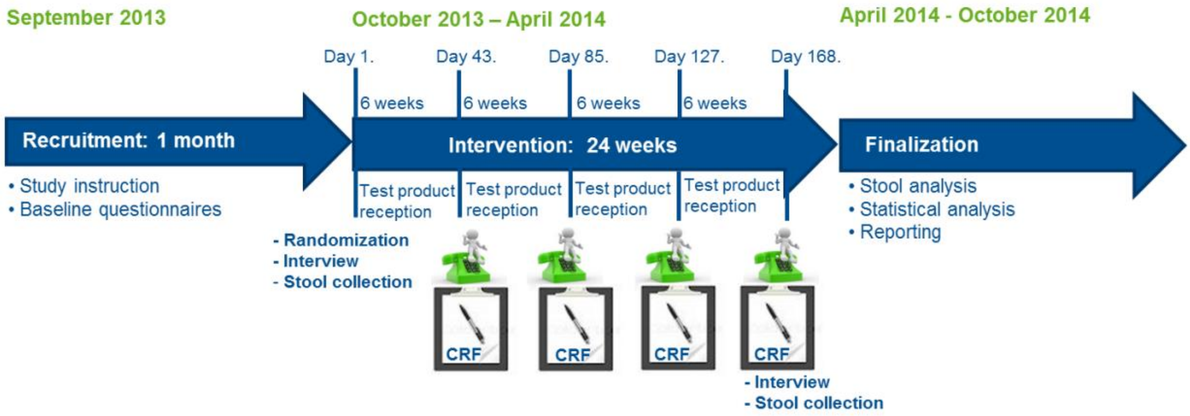

Supplemental Figure 1. Overview of the study design of the randomized controlled double-blind explorative study investigating the effect of prebiotic inulin-type fructans on acute infectious diseases in children
